# Genome-wide association mapping for root traits associated with frost tolerance in faba beans using KASP-SNP markers

**DOI:** 10.3389/fgene.2022.907267

**Published:** 2022-08-24

**Authors:** Ahmed Sallam, Yasser S. Moursi, Regina Martsch, Shamseldeen Eltaher

**Affiliations:** ^1^ Department Genebank, Leibniz Institute of Plant Genetics and Crop Plant Research (IPK), Gatersleben, Germany; ^2^ Department of Genetics, Faculty of Agriculture, Assiut University, Assiut, Egypt; ^3^ Department of Botany, Faculty of Science, Fayoum University, Fayoum, Egypt; ^4^ Department of Crop Sciences, Georg-August-Universität Göttingen, Göttingen, Germany; ^5^ Department of Plant Biotechnology, Genetic Engineering and Biotechnology Research Institute (GEBRI), University of Sadat City (USC), Sadat, Egypt

**Keywords:** *Vicia faba*, KASP, freezing temperature, root traits, GWAS

## Abstract

Frost is an abiotic stress factor that threatens plant development and crop productivity not only in cold regions but also in temperate zones. Roots play an important role in plant growth during frost stress. Therefore, variation in root characteristics could be studied to improve frost tolerance in winter faba bean. The present study aimed to identify the genomic regions that control frost tolerance in a winter faba bean population by focusing on root-related traits. A set of 185 genotypes were tested for frost tolerance under artificial frost growth conditions at −16°C, −18°C, and −19°C in a growth chamber. Frost stress reduced the root-related parameters in all genotypes, with a wide variation among genotypes. A genome-wide association study identified nine novel single-nucleotide polymorphisms that are associated with the root-related traits. The most frost-tolerant genotypes were identified; two genotypes, S_028 and S_220, exhibited remarkable performance under frost stress. Moreover, they harbored all four of the alleles favorable for frost tolerance. Remarkably, two markers showed genetic pleiotropic effects with positive allele effects on root fresh matter and root dry matter. Thus, both genotypes can be implemented in a breeding program to provide the alleles for healthier roots under frost conditions to develop more frost-tolerant varieties, and the two markers can be used to screen large collections to select for frost tolerance. These results may provide novel insights for improving frost tolerance in faba beans and in other legume crops.

## Introduction

The faba bean (*Vicia faba* L.) is the only edible pulse crop among *Vicia* species (Lyu et al., 2021). Relative to other grain legumes, the faba bean is ranked fourth after chickpea, field pea, and lentil (Rebaa et al., 2017; Alharbi and Adhikari, 2020), with a global production of approximately 4.5 million tons produced from a cultivated area of approximately 2.5 Mha (Food and agriculture organization of the United Nations, 2019). The high protein content and well-balanced amino acid profile of the faba bean distinguish it from other legumes (Roy et al., 2010; Lyu et al., 2021). Owing to its high protein content, the faba bean is grown as a food crop in developing countries but principally as a livestock feed in Europe (Sallam et al., 2016a; Nadeem, 2021). Faba bean are less likely to generate off-flavors than soybean and pea owing to their low lipid content and low endogenous lipoxygenase activity, allowing them to be incorporated into everyday meals (Chang and McCurdy, 1985). As the demand for organic food grows, legume-based crops should be emphasized in crop rotations alongside cereals and oilseeds. Similar to other leguminous plants, the faba bean crop fixes atmospheric nitrogen into biologically usable ammonia, facilitating natural nitrogen fertilization of soils (Barton et al., 2014). Another advantage of the faba bean over other legumes is its ability to adapt to a wide range of climatic and soil conditions (Pociecha et al., 2008; Katerji et al., 2011; Zhou et al., 2018). Nevertheless, faba bean are also susceptible to a range of biotic and abiotic challenges, which contribute to loss due to environmental factors, including frost (Sallam et al., 2016b; O’Sullivan and Angra, 2016).

Frost is an important climatic factor that affects agricultural production and the agroforestry economy in temperate and subtropical areas worldwide (Snyder and Melo-abreu, 2005; Papagiannaki et al., 2014; Ambroise et al., 2020). Moreover, because of global warming and climate change, it is predicted that frost will become more problematic (Gu et al., 2008). In Northern and Central Europe, severe winter frost is a major abiotic stress factor affecting beans, and owing to the insufficient winter hardiness of the genotypes in use, the faba bean is primarily cultivated as a spring crop (Arbaoui et al., 2008; Sallam et al., 2016b). Frost stress not only decreases productivity but also lowers the diet value as a result of seed staining (Hawthorne, 2007). Additionally, frost can destroy the N-fixing bacteria by reducing the activity of nitrogenase, which threatens the symbiotic relationship (Stoddard et al., 2006).

To avoid the severe effects of summer drought on grain filling and ultimate yield, farmers have to sow the crops earlier and thus seek more frost-tolerant varieties (Rezaei et al., 2015). Although winter-type faba beans have a higher yield and protein content than spring-type faba beans, they are primarily grown as a spring crop in cool-temperate locations (Arbaoui et al., 2008; Sallam et al., 2015; Sallam et al., 2016a). Therefore, breeding for improving frost tolerance and developing cultivars with high frost tolerance is urgently needed. The development of frost-tolerant varieties *via* conventional breeding is challenging as frost tolerance is a polygenic trait (Li et al., 2011; Avia et al., 2013; Sallam et al., 2016a). Three parameters are critical in determining a genotype’s winter hardiness: (i) frost tolerance, (ii) ability to survive biotic stresses such as snow mold, and (iii) resistance to abiotic stress factors such as high water saturation in the soil (Arbaoui, 2007; Sallam and Martsch, 2015; Sallam et al., 2016b). Thus, in addition to testing under simulated frost growth conditions in growth chambers, faba bean genotypes should be tested in the field to determine the overall winter hardiness of the most tolerant genotypes. In multi-location trials, many traits associated with winter hardiness should be assessed and analyzed. According to the climate prediction models, frost conditions will become more severe, causing a marked reduction in plant production due to a lack of root growth (Ambroise et al., 2020). Most studies on frost tolerance have focused on the examination of the aboveground organs; this is attributed to the fact that roots are protected under the soil surface (Lee et al., 2009; Smékalová et al., 2014). A previous study showed that, at the seedling stage, the faba bean is considered less cold tolerant than its wild relatives belonging to the western Mediterranean regions (Inci and Toker, 2011). Recently, Alharbi et al. (2021) have studied the effects of frost on pod setting in faba bean under field conditions, assessing several traits at the vegetative and reproductive stages. In both studies, the authors evaluated several morphological and yield traits but no root traits.

The faba bean is a diploid plant with 2n = 2x = 12 chromosomes and has one of the largest genomes among crops (approximately 13,000 Mb) (Satovic et al., 2013) compared with the genomes in soybean (1,200 Mb) and in field pea (4,000 Mb) (Torres et al., 2006; Satovic et al., 2013). This may make physical and genetic mapping, as well as map-based cloning, more challenging (Ellwood et al., 2008; Lavania et al., 2015). Because of the complexity of the faba bean genome, it is difficult to find a reference genome that can be used for genotyping by sequencing (GBS), for example, other crops such as wheat (*Triticum aestivum*), barley (*Hordeum vulgare*), maize (*Zea mays*) and rice (*Oryza sativa*). Webb et al. (2016) constructed a faba bean consensus map (FBCM) using single-nucleotide polymorphism (SNP) markers developed from *Medicago truncatula* (legume model). The SNPs in this map were used in many studies to identify alleles associated with the target traits in the faba bean.

Using the recent advances in molecular genetics tools and methods along with a breeding program will accelerate the genetic improvement in frost tolerance in faba bean. One of these tools, marker-assisted selection (MAS), has been identified as a powerful approach for accelerating breeding operations to improve certain traits. Association mapping is one of the most successful strategies for identifying quantitative trait loci (QTLs) that underlie trait variation among the different methods used for MAS. Moreover, it is a tool for mapping QTLs that regulate complex characteristics on the basis of marker–trait linkage disequilibrium. Previously, a genome-wide association study (GWAS) has been successfully employed to identify causative alleles associated with frost stress in faba bean. The GWAS can be conducted using any type of DNA molecular marker. Recently, kompetitive allele-specific PCR (KASP) genotyping has been introduced by LGC limited to be applied in QTL mapping and the GWAS (Semagn et al., 2014). The KASP is designed for a single-nucleotide polymorphism which differs between genotypes of the same species. KASP markers have many advantages over other DNA molecular markers including high sensitivity, reliability, reproducibility, and genotyping accuracy (Thomson, 2014). Webb et al. (2016) produced a set of 757 KASP markers that were used to genotype many faba bean populations for GWAS and QTL mapping studies.

Several studies have been published on frost tolerance in faba bean plants using the same genotypes, and several morphological characteristics such as stem and leaf characteristics, as well as physiological characteristics, have been studied (Sallam and Martsch, 2015; Sallam et al., 2015; Sallam et al., 2016a; Sallam et al., 2016b). Likewise, using the GWAS, alleles associated with several morphological parameters including loss of color and turgidity, freezing survival, and regrowth after freezing have been identified in the same collection of genotypes (Ali et al., 2016).

The effects of frost on root traits have not been extensively studied, and our study is one of the first reports, to the best of our knowledge, to investigate this essential issue. These screenings focused on the morphological traits of faba bean roots, such as root length (RL), root frost susceptibility (RFS), root fresh matter (RFM), and root dry matter (RDM). Thus, the present study aimed to i) investigate the genetic variation in root traits under frost stress; ii) study the correlations between frost tolerance and root traits; and iii) identify marker–trait associations (MTAs) and QTLs associated with root traits.

## Materials and methods

A set of 183 single-seed-descent (SSD) lines of the faba bean from the Göttingen Winter Bean Population (GWBP) were used as plant materials (Supplementary Table S1). These SSD lines were developed from eleven founder lines: six lines from Germany (Webo, Wibo, Hiverna/1, 79/79, L977/88, and L979/S1), two lines from France (Côte d’Or/1 and Arrisot), and three lines from the UK (Banner, Bourdon, and Bulldo). These 11 founder lines were first sown in open pollination conditions and natural selection in order to produce a freely recombining population. After eight generations of ongoing maintenance of this population, < 400 SSD were drawn and inbred to generation > F9 and beyond (Stelling, 1989; unpublished; Gasim, 2003).

Two additional spring faba bean lines were used as controls (checks) in the frost experiments: Hedin/2 and Minica (Sallam and Martsch, 2015; Sallam et al., 2015; Sallam et al., 2016b).

### Experimental layout and artificial frost experiment

The 185 genotypes (183 SSD lines + 2 controls) were assessed for frost tolerance in a Vötsch frost growth chamber (FGCh; 4 m^2^). All genotypes were assessed for frost tolerance in eight replications. The use of the 4 m^2^ area of this chamber once corresponded to one replication. Each replication included all genotypes in an alpha lattice design. In each replication, all genotypes were sown in pots filled with a mixture of compost soil and sand (3:1, respectively). Each pot contained four different genotypes with two seeds per genotype (Supplementary Figure S1). When all plants reached the two-leaf stage (at room temperature), all pots were moved to the well-isolated FGCh. The growth conditions (program) in the FGCh were set at a light exposure of 200 μmol s^−1^ m^−2^ for 10 h per day and 80–90% humidity. The seedlings of all genotypes were exposed to two phases described as follows: (I) a hardening phase in which all genotypes were exposed to a temperature of 4°C during the day and 0°C at night for 10 days and (II) the true frost test for 3 days at −16°C, −18°C, and −19°C, respectively, during the night and thawing during the hardening and frost days (Supplementary Figure S2). Plants were irrigated only during the hardening phase to keep the pots at approximately 70% of the soil’s water capacity.

### Scoring and measurement of phenotypic traits

In a previous study by Sallam et al. (2015), shoot traits in a set of 216 genotypes, including our 185 genotypes, were scored: loss of leaf color + loss of leaf turgidity (LC + LT) and frost tolerance index (FTI). The low values of LC + LT and the high values of FTI indicated frost tolerance according to Sallam et al. (2015). In addition to the shoot traits, this study also assessed the root traits but did not report them. In the present study, we evaluated the root traits and studied their relationship with frost tolerance.

**FIGURE 1:**
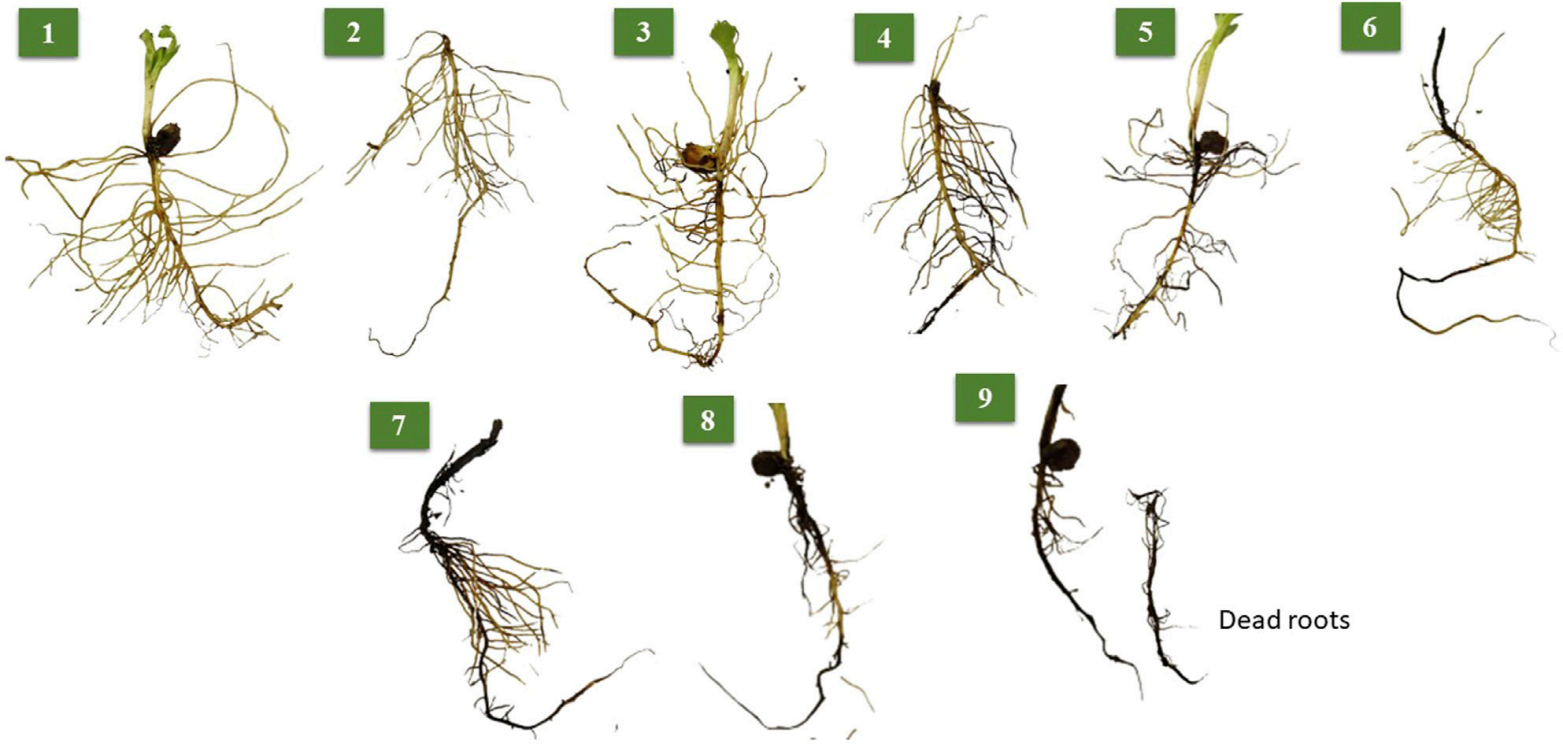
Visual scoring of root frost susceptibly scored on the 189 genotypes.

After the frost test, the soil in each pot was carefully washed and removed to avoid root damage. Then, the roots of plants belonging to each genotype were cautiously washed again and prepared for the assessment of four root traits. RFS was visually scored from 1 (healthy roots) to 9 (withered black dead roots; Figure 1). RL was measured in cm, whereas RFM and RDM were measured in grams. Lower values of RFS and higher values of RL, RFM, and RDM indicated tolerance to frost.

### Statistical analyses for root traits

The analysis of variance was performed with PLABSTAT software (Utz, 1997) using the following equation:

$${\text{Y}}_{ij}=\mu +{\text{g}}_{i}+{\text{r}}_{j}+{\text{g}}_{rij},$$

where Y_*ij*_ is the observation of the genotype *i* in replication *j*; μ is the general mean; g_*i*_ and r_*j*_ are the main effects of the genotype and replication, respectively; g_*rij*_ is the genotype × replication interaction of the genotype *i* with replication *j*. Broad-sense heritability (*H* ^2^) of each trait was calculated as the ratio of the genotypic variance with the phenotypic variance: *H* ^2^ = σ^2^ *g*/σ^2^ *p* (Rasmusson and Lambert, 1960). Pearson’s correlation coefficient was calculated among all traits by PLABSTAT software (Utz, 1997).

### DNA extraction and KASP genotyping

Using Illustra Nucleon Phytopure Genomic DNA Extraction kits (GE Healthcare Life Sciences, UK), DNA was isolated from the 11 founder lines and GWBP. The DNA was genotyped utilizing the KASPar™ (kompetitive allele-specific PCR) test platform (KBioscience, United Kingdom), a single-plex SNP genotyping technology that uses allele-specific amplification followed by fluorescence detection. Of a total of 687 SNPs identified in the FBCM developed by Webb et al. (2016), 189 SNP markers were polymorphic among the 11 founder lines and were chosen to genotype the 189 genotypes (Sallam et al., 2016a; Webb et al., 2016). These SNP markers were mapped to six linkage groups using the legume model*, Medicago truncatula*. Each linkage group corresponded to one chromosome. The list of 189 SNPs and the sequence of KASP markers are presented in Supplementary Table S2.

### Population structure and GWAS

Population structure analysis was performed using the 189 KASP markers on the whole population (Sallam and Martsch, 2015; Sallam et al., 2016a). The analysis revealed no population structure among the genotypes. Therefore, a GWAS was performed between markers and root traits using a general linear model (GLM). Significant markers associated with the root traits were detected using a threshold of 1% Bonferroni correction (Duggal et al., 2008). The GWAS was performed by TASSEL v5.2.40 (Bradbury et al., 2007). The phenotypic variation explained by marker (*R* ^2^) and allele effects was estimated for each trait by TASSEL v5.2.40. The quantile–quantile (QQ) plot was presented for each trait using the R package “qqman” (Turner, 2018). MapChart v2.2 (Voorrips, 2002) was used to illustrate the QTLs and their positions in the linkage group in the FBCM. The linkage disequilibrium (*r* ^*2*^) was calculated between each pair of significant SNPs located on the same chromosome by TASSEL v5.0.

## Results

### Genetic variation

**FIGURE 2:**
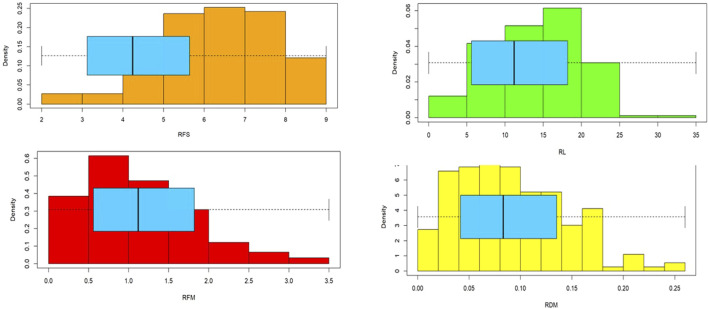
Histogram showing the distribution of all genotypes in each trait and box plot analysis illustrating the minimum, maximum, and mean values for each trait.

All the spring genotypes died under the imposed frost stress. Highly significant differences were found among all genotypes for all root traits scored after frost stress (Table 1). The H^2^ values were 0.78, 0.74, 0.70, and 0.66 for RFS, RL, RDM, and RFM, respectively. The minimum, maximum, and mean values for all genotypes are presented in Table 1. The distribution of all genotypes for all traits is presented in Figure 2. Each trait had a wide range: 2.06–9.00 for RFS, 1.94–30.88 cm for RL, 0.11–3.44 g for RFM, and 0.01–0.26 g for RDM (Table 1; Figure 2). RFM and RDM exhibited a right-skewed distribution, whereas RFS had a left-skewed distribution. RL tended to have a normal distribution with some skew. The most tolerant genotype differed by traits. The S_002 genotype was the most frost-tolerant genotype with respect to RFS, with a score of 2.06. The genotype S_132 showed the highest RL of 30.88 cm. S_028 and S_052 genotypes showed the highest RFM and RDM, respectively.

**TABLE 1:** Ranges including minimum, maximum, and mean values, least-square difference, F-value, and heritability estimates (*H* ^2^) for root length, root fresh susceptibility, root fresh matter, and root dry matter of faba bean genotypes evaluated under frost conditions.

	Minimum	Maximum	Mean	L.S.D (*p* = 0.05)	F-value	Heritability
Root fresh susceptibility	2.06	9.00	6.36	1.83	4.59^∗^^∗^	78.20
Root length	1.94	30.88	14.33	8.30	3.90^∗^^∗^	74.37
Root fresh matter	0.11	3.44	1.13	1.11	2.93^∗^^∗^	65.82
Root dry matter	0.01	0.26	0.096???	0.08	3.31^∗^^∗^	69.81

F-value shows the genetic variation among genotypes. ^∗^^∗^ *p* < 0.01.

### Phenotypic correlation

**FIGURE 3:**
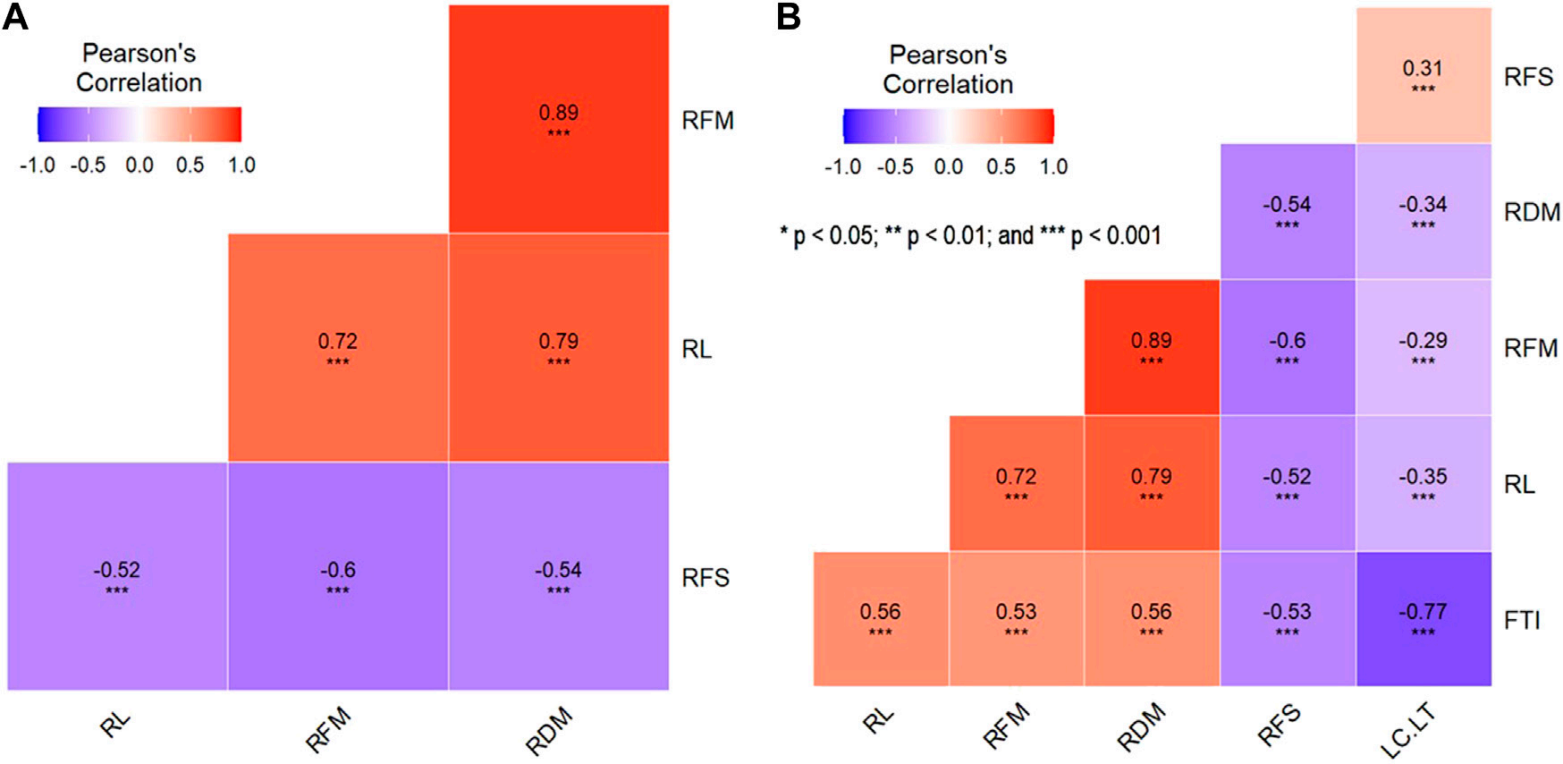
**(A)** Phenotypic correlation analysis among root traits and **(B)** correlation between both LC + LT and FTI scored on seedling shoots and root traits.

Pearson’s correlation analysis revealed very high and significant correlations among root traits (Figure 3A). A significant positive correlation was found among RL, RFM, and RDM. The highest correlation was found between RFM and RDM (*r* = 0.89^∗^^∗^^∗^). RFS was negatively and significantly correlated with the other root traits, with *r* = −0.54^∗^^∗^, −0.52^∗^^∗^, and −0.60^∗^^∗^ between RFS and RDM, RL, and RFM, respectively.

As mentioned in the materials and methods, the FTI and LC + LT scored by Sallam et al. (2015) were used in this study as key frost-tolerant traits to test their correlation with root traits (Figure 3B). The correlation between root traits and both FTI and LC + LT is presented in Figure 3B. A significant negative correlation was found between LC + LT and RL, RDM, and RFM, whereas a significant positive correlation was found between LC + LT and RFS (*r* = 0.31^∗^^∗^).

The FTI was significantly correlated with all root traits; it was positively and significantly correlated with RDM (*r* = 0.56^∗^^∗^^∗^), RFM (*r* = 0.53^∗^^∗^^∗^), and RL (*r* = 0.56^∗^^∗^^∗^) and negatively and significantly correlated with RFS (*r* = −0.53^∗^^∗^).

### Genome-wide association mapping

**FIGURE 4:**
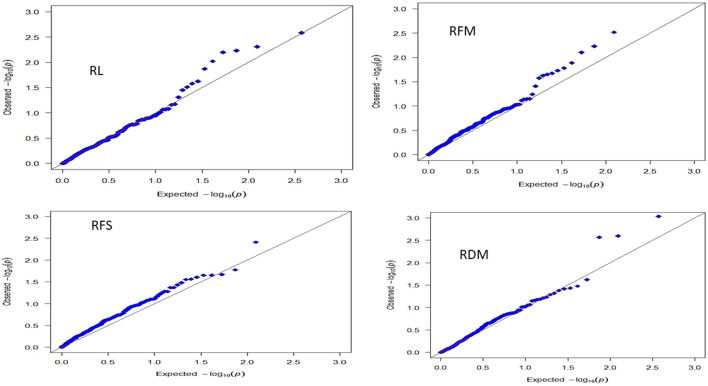
QQ plots resulted from the GWAS using the GLM model for each trait.

A marker–trait association analysis was performed between the 189 SNP markers and root traits for all genotypes (Table 2). Nine SNPs were found to be significantly associated with all root traits. The Manhattan plot of all the nine significant SNPs is presented in Supplementary Figure S3. The significant markers were distributed on chr1, chr3, chr5, and chr6. Chromosome 3 had the highest number of QTLs (*n* = 4), followed by chromosome 5 with three QTLs. A single QTL was identified on chromosomes 1 and 6. Of the nine SNP markers, eight markers had positive allele effects (i.e., enhancing the respective trait), but Vf_Mt4g091530_001 showed a negative allele effect on RFS (i.e., reducing the respective trait; Table 2). The resultant QQ plots for each trait are presented in Figure 4.

**TABLE 2:** List of significant SNPs associated with root traits using the GLM model including the *p*-value, chromosome number, phenotypic variation, target allele, and allele effect.

Trait	Marker	*p*-value	Chromosome no.	Position	Marker *R* ^2^%	Allele	Allele effect
RL	Vf_Mt1g072640_001	0.00496	3	67.73	7.34	C	3.34
RL	Vf_Mt7g051360_001	0.00261	5	33.55	6.75	A	3.32
RFS	Vf_Mt4g091530_001	0.00389	6	90.81	5.81	A	−0.78
RFS	Vf_Mt5g009720_001	0.000439	1	77.56	4.89	G	−0.84
RFM	Vf_Mt1g072640_001	0.000159	3	67.73	4.87	C	0.51
RFM	Vf_Mt7g073970_001	0.00304	5	62.92	4.78	C	0.31
RDM	Vf_Mt1g072640_001	0.000928	3	67.73	4.64	C	0.035
RDM	Vf_Mt1g082210_001	0.0027	3	83.25	4.49	A	0.029
RDM	Vf_Mt7g073970_001	0.00252	5	62.92	4.21	C	0.024

Two SNPs (Vf_Mt1g072640_001 and Vf_Mt7g051360_001) located on chromosomes 3 and 5, respectively, were found to be significantly associated with RL. The phenotypic variation explained (*R* ^2^) was 7.34 and 6.75% for the SNP markers Vf_Mt1g072640_001 and Vf_Mt7g051360_001, respectively. The target allele (C) of Vf_Mt1g072640_001 and allele (A) of Vf_Mt7g051360_001 had allele effects of 3.34 and 3.32, respectively, on RL (Table 2).

Similarly, two SNPs (Vf_Mt4g091530_001 and Vf_Mt5g009720_001) located on chromosomes 6 and 1, respectively, were found to be significantly associated with RFS. *R* ^2^ was 5.81 and 4.89% for Vf_Mt4g091530_001 and Vf_Mt5g009720_001 markers, respectively. The target allele (A) of Vf_Mt4g091530_001 had a negative allele effect of −0.78 on RFS, whereas the target allele (G) of Vf_Mt5g009720_001 had an allele effect of −0.84 on RFS (Table 2).

Vf_Mt1g072640_001 and Vf_Mt7g073970_001 were found to be significantly associated with RFM. The phenotypic variation explained *R* ^2^ was 4.87 and 4.78% for Vf_Mt1g072640_001 and Vf_Mt7g073970_001 markers, respectively. The target allele (C) of Vf_Mt1g072640_001 and allele (C) of Vf_Mt7g073970_001 had allele effects of 0.51 and 0.31 on RFM, respectively (Table 2).

Three SNPs (Vf_Mt1g072640_001, Vf_Mt1g082210_001, and Vf_Mt7g073970_001) were found to be significantly associated with RDM. *R* ^2^ was 4.64, 4.49, and 4.21% for Vf_Mt1g072640_001, Vf_Mt1g082210_001, and Vf_Mt7g073970_001, respectively. The target alleles of these three markers had positive allele effects. The target alleles of Vf_Mt1g072640_001 (C), Vf_Mt1g082210_001 (A), and Vf_Mt7g073970_001 (C) had allele effects of 0.035, 0.029, and 0.024 on RDM, respectively (Table 2).

**FIGURE 5:**
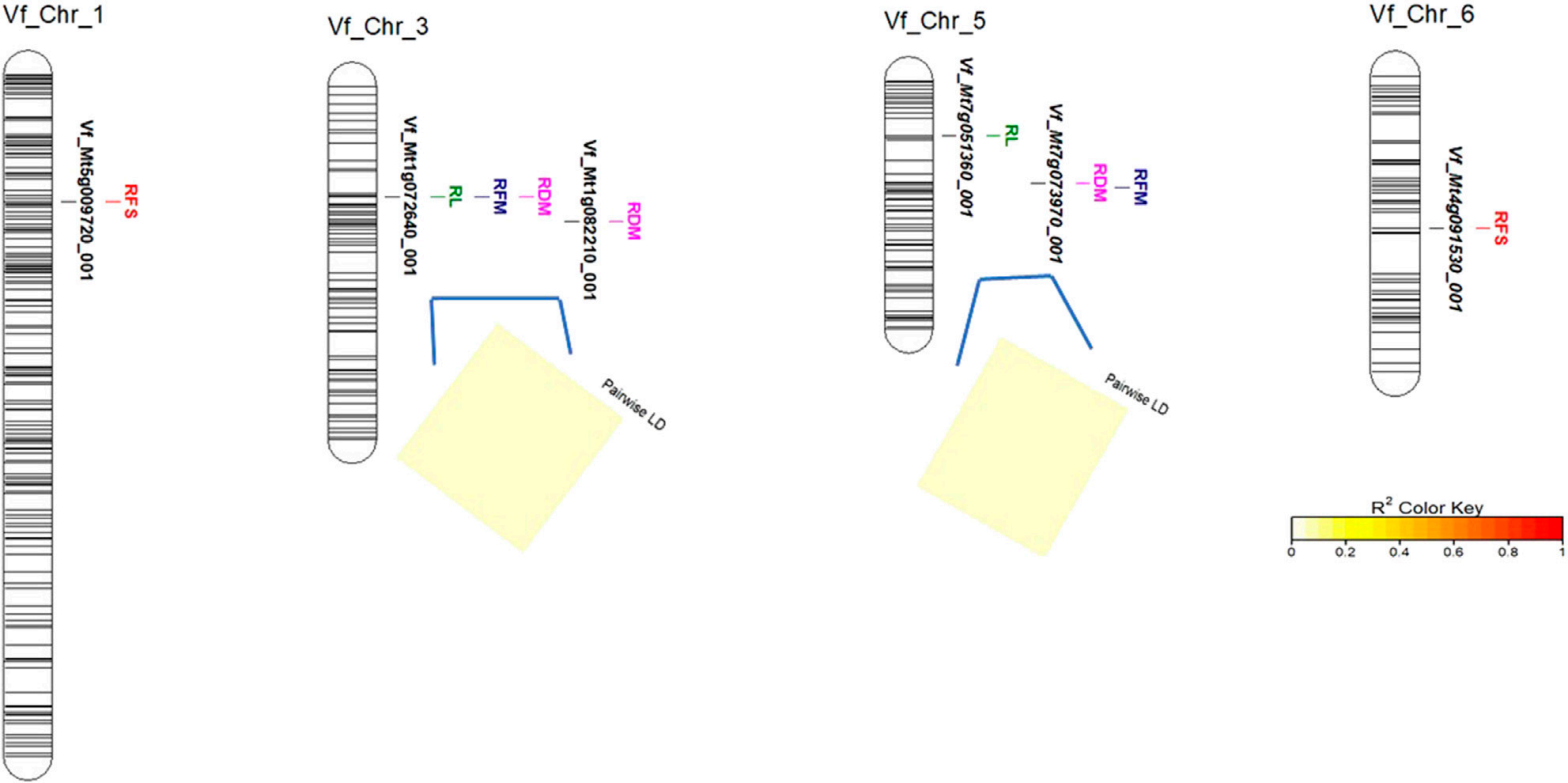
Distribution of QTLs associated with root traits detected by the GWAS and LD analysis (*r* ^2^) between SNPs located on the same chromosome.

The linkage disequilibrium (LD; *r* ^2^) between each SNP pair located on the same chromosome was tested. No significant LD was found between markers located on chromosome 3 or 6 (Figure 5).

Notably, two markers showed a kind of pleiotropy as each marker was associated with more than one trait. The Vf_Mt1g072640_001 marker was found to be significantly associated with RL, RFM, and RDM with positive allele effects on all these traits, and Vf_Mt7g073970_001 had a significant association with RDM and RFM.

## Discussion

### Genetic variation

The high genetic variation among genotypes in the GWBP for all root traits is valuable for the improvement in frost tolerance in winter faba beans. The genetic variation was very useful for discriminating between the tolerant and susceptible genotypes in each trait. The high genetic variation among genotypes for all traits was expected as the population was derived from a natural cross of eight founder lines. This high genetic variation makes the GWBP ideal for various studies. The same population was used for studying frost tolerance on shoots at the seedling stage (Sallam et al., 2015), winter hardiness (Sallam et al., 2016a), drought tolerance (Ali et al., 2016), and agronomic performance (Gasim and Link, 2007).

In this study, all plants of the two spring control lines died during frost stress, indicating that the freezing temperature used in this study was suitable to determine the true performance of each genotype for each trait, that is, to distinguish the tolerant genotypes from the susceptible ones. The 10 most frost-tolerant genotypes for each trait were determined. Only two genotypes, S_028 and S_220, were found to be common among the 10 genotypes most tolerant with respect to the four root traits. These two genotypes experienced less injury from frost stress (low RFS score) and showed the highest values of RL, RFM, and RDM. Moreover, the two genotypes had been previously identified as frost-tolerant genotypes for shoot traits and had high survival after frost (Sallam et al., 2015). Notably, Sallam et al. (2016a) reported the same two genotypes with high winter survival under natural freezing temperatures in field conditions (high winter hardiness).

As yield is the ultimate goal of breeding programs and the aboveground organs experience higher frost stress than roots also because studying roots in the soil in field conditions is challenging. Thus, most studies on frost resistance have focused on the aboveground organs; however, roots play a crucial role in plant growth and final productivity. Roots need to stay biologically active for a longer time than the aboveground organs to ensure sufficient water and mineral supply to avoid reduction in plant growth and productivity (Ryyppö et al., 1998). Belowground frost damages fine roots, which are important for water and mineral uptake; regeneration of these damaged fine roots would occur at the expense of the shoot (Gaul et al., 2008). Root development was demonstrated to play a key role in the survival and establishment of sorghum grown under chilling conditions (Bekele et al., 2014). This observation is in agreement with our findings as frost damage to fine roots, particularly in the susceptible genotypes, results in plant death. Studying root traits at the seedling stage may provide good and useful information on the root characteristics of each genotype as roots can be easily removed from the pot and scored.

RFS, an important trait related to frost tolerance in faba beans, signifies the effect of frost on roots (Figure 1). In the present study, many genotypes, including the spring genotypes, showed withered black roots after the frost test. Similarly, a visual screening scale for the aboveground organs has been successfully used to select for frost tolerance in faba beans. The most tolerant genotypes showed less injury, whereas the most susceptible genotypes had withered black leaves and stems (Inci and Toker, 2011). It has been reported that the roots of susceptible genotypes did not die during the frost period but rather were affected during the cold acclimation period (Ambroise et al., 2020). This trait was studied by Sallam et al. (2015), but an analysis of other root traits such as RL, RFM, and RDM under frost stress was reported for the first time in the present study to the best of our knowledge. The high heritability estimates for all traits were due to the non-significant environment. Such high heritability estimates indicate that selecting root traits to improve frost tolerance in a breeding program is feasible and effective.

### Phenotypic correlation

Highly significant phenotypic correlations were found among all root traits under frost stress (Figures 3A, B). This result indicates the possibility of improving these traits together through a breeding program by selecting the best genotypes for all four root traits. Similar results were reported in sorghum under chilling conditions; root traits showed significant positive correlations under both normal and chilling conditions (Bekele et al., 2014). RFS was found to be negatively correlated with all other root traits, indicating that the less the frost injury in roots, the longer the roots (RL) and the higher the fresh and dry matter yield (RFM and RDM; Figures 3A, B). Kreyling et al. (2012) reported that freezing temperature causes severe damage to fine roots, resulting in a significant decrease in root biomass (by 50%). Similarly, a negative correlation was found between the total root density and frost susceptibility (Laughlin et al., 2021). Moreover, they found that plants with longer roots were found to tolerate frost better than those with shorter roots (Laughlin et al., 2021). These findings are consistent with our results as a high negative correlation was found between RFS and RFM (r = −0.60^∗^^∗^). To understand the interplay between root traits and aboveground organ (shoot and leaf) traits that were scored earlier in the same population by Sallam et al. (2015), a correlation analysis among these traits was performed. LC + LT refers to the symptoms of frost stress on the leaves of faba bean seedlings, whereas the FTI comprises survival traits (regrowth after frost and tendency to survive) scored after frost (Sallam et al., 2015). The highly significant phenotypic correlation between shoot and root traits highlighted the importance of healthy roots in frost tolerance. The correlation of FTI with all root traits showed a higher significance than LC + LT with all root traits, indicating the role of roots in the survival mechanism after the frost test (Figure 3B). However, shoots exhibited different physiological aspects and stress responses than those exhibited by roots, indicating two different frost hardening and resistance mechanisms (Ryyppö et al., 1998). In the present study, highly significant positive correlations were found between shoot and root traits. Likewise, in sorghum, under chilling conditions, root biomass and RL were positively correlated with plant survival (Balota et al., 2010; Bekele et al., 2014). Similarly, root weight has been used successfully as a selection parameter for cold tolerance in Cyprus Vetch under field growth conditions (Ratinam et al., 1994). Taken together, these findings suggested that it is crucial for plants to have highly biologically active roots for a longer time than any other organs (e.g., shoots) as the roots are the main source of water and nutrient supply to aboveground tissues.

Therefore, breeding for root frost tolerance at the seedling stage is very important to produce cultivars with high frost tolerance. It has been previously reported that there is a significant correlation between frost tolerance at the seedling stage and under field conditions (Sallam et al., 2016a; Sallam et al., 2017). Because it is difficult to study the roots in field conditions, studying the root traits at the seedling stage is a suitable alternative to obtain information on the degree of frost tolerance of each tested genotype.

### Genome-wide association

Association mapping is regarded as a powerful approach for identifying variations responsible for key features in various crops (Pritchard and Przeworski, 2001; Reich et al., 2001). The association mapping in GWBP was performed on various genetic backgrounds of 189 SSD lines obtained from a natural cross between 11 genotypes collected from different parts of Europe (Sallam et al., 2015). A GWAS using the same number of SNPs (189) has been performed on a subset of this population (*n* = 182 genotypes) in earlier studies to identify alleles associated with frost tolerance in faba bean shoots (Sallam and Martsch, 2015; Sallam et al., 2016b) and determine leaf fatty acid composition under hardening conditions (Sallam et al., 2016b), winter hardiness in field conditions (Sallam et al., 2016a), drought tolerance at the seedling stage (Ali et al., 2016), and *Ascochyta fabae* resistance (Faridi et al., 2021). These studies indicate that this population has a wide genetic variation, which allows breeders and geneticists to identify markers associated with the target traits. However, in the present study, the number of KASP markers was lower than that in other GWAS studies in other crops because of the higher complexity of the faba bean genome relative to that of other legume crops and the lack of a faba bean reference genome. Thus, genotyping by sequencing in faba beans remains a challenging task. The KASP markers used in the present study may be the best option for genetic studies to identify markers associated with target traits in faba beans (Sallam and Ul-Allah, 2019). This set of KASP markers was initially developed by Webb et al. (2016). The GWBP is a highly diverse population ideal for the GWAS because (1) it has no population structure, which causes spurious association, and (2) it has an extremely low degree of LD (Sallam et al., 2015). A long-range LD increases the number of false associations in the marker–trait association analysis by the GWAS (Alqudah et al., 2020).

As there is no population structure among genotypes in the GWBP, a GLM model was the appropriate analysis approach that fits with association mapping (Alqudah et al., 2020). The results of QQ plots resulting from the GWAS for each trait further supported the efficiency of the GLM model in detecting the marker–trait associations. Most of the observed and expected *p*-values lie on the diagonal line except for the true significant markers associated with the traits. The QQ plot analysis indicates the efficiency of GWAS results (Alqudah et al., 2020).

In the present study, the association analysis revealed that nine different significant markers were associated with RL, RFS, RFM, and RDM (Table 2; Figure 5). In the same population, 52 SNPs associated with frost tolerance in faba bean shoots at the seedling stage were previously reported (Sallam et al., 2015). Using a biparental winter faba bean population (101 recombinant inbred lines) and a set of 113 KASP markers developed by Webb et al. (2016), 27 QTLs were found to be associated with frost tolerance-related shoot traits at the seedling stage (Sallam et al., 2016b). Thus, the present study reports novel SNPs associated with root traits under frost stress. All SNPs detected in the two aforementioned studies were located on chromosomes 1, 2, 3, 4, and 6 in the FBCM (Webb et al., 2016). Most of the SNPs associated with frost tolerance in the shoots were located on chromosome 1 (Sallam and Ul-Allah, 2019). In the present study, the nine identified SNPs were located on chromosomes 1, 3, 5, and 6. Notably, two novel SNPs associated with root traits were located on chromosome 5, which had not been previously reported to be associated with shoot traits either in the GWBP or the biparental populations.

Remarkably, when the same KSAP markers were used to identify the markers associated with frost tolerance in the shoots of two different populations, no marker was found to be significantly associated with any of the shoot traits. This finding indicates that the markers significantly associated with root traits were entirely different from those associated with shoot traits under frost stress. This also supports the idea that the mechanism of frost resistance in the leaves or shoots is different from that in the roots. This is partially supported by the negative and significant correlations between the leaf traits (LC + LT) and the root traits (RL, RFM, and RDM; Figure 3B).

Notably, two markers were found to be associated with more than one trait: Vf_Mt1g072640_001 associated with RL, RFM, and RDM and Vf_Mt7g073970_001 associated with RFM and RDM (Table 2; Figure 5). It is important to note that these two markers had pleiotropic genetic effects (i.e., for each marker, the same allele controls the variation in both traits RFM and RDM simultaneously), and their alleles have positive allele effects. It is highly likely that the alleles of these two markers play a crucial role in frost tolerance by increasing these advantageous traits. Another valuable marker is Vf_Mt4g091530_001, which had a negative allele effect on RFS, indicating that it increases frost tolerance by decreasing RFS and should be validated in different genetic backgrounds to be used for improving RFM and RDM because the allele effects for both markers (Vf_Mt1g072640_001 and Vf_Mt7g073970_001) were positive. Moreover, all markers detected in this study had *R* ^2^ < 0.10, which indicated that all these QTLs had minor effects on frost tolerance. Non-significant LD between marker pairs located on chromosomes 3 and 5 indicates that these SNPs represent individual QTL associated with root frost tolerance.

Notably, the two markers associated with RL, RFM, and RDM could be considered very informative markers as they had pleiotropic genetic effects (Table 2; Figure 5), and their target alleles have positive allele effects on these traits. Genome-wide association study was very useful to identify alllele associated with root traits under frost stress. However, more studies are need to understand the genetic control of frost tolerance in faba bean. Moreover, genomic selection in fabe bean should be utilized to improve target trait such as frost tolerance. Genomic selection is the best approach for breeding target traits after considering seed amount, cost, labor and time (Sandhu et al., 2021).

## Conclusion

This study elucidates the vital role of roots and their association with frost tolerance in winter faba beans. Nine novel SNPs and genomic regions controlling root traits under frost stress were reported. The highly significant correlations found between the FTI and root traits are promising for the selection of truly frost-tolerant genotypes. The two genotypes S_028 and S_220 are candidate parent genotypes because both of them show tolerance to frost stress at the level of shoots and roots at the seedling stage and winter hardiness under field conditions. Notably, both genotypes possess four alleles out of the six frost tolerance-associated alleles. The two markers Vf_Mt1g072640_001 and Vf_Mt7g073970_001 are of high significance because they show pleiotropic effects on RFM and RDM, and their alleles have positive additive effects on both traits. Notably, the allele C of the marker Vf_Mt1g072640_001 can be used to select for improving RL, RFM, and RDM, and the allele A of the marker Vf_Mt4g091530_001 decreases RFS. Thus, employing these two genotypes and these markers may help in developing or selecting faba bean cultivars with high frost tolerance and high winter hardiness.

## Data Availability

The original contributions presented in the study are included in the article/Supplementary Material; further inquiries can be directed to the corresponding author.
