# Supplementary material for: Genome-wide association mapping for root traits associated with frost tolerance in faba beans using KASP-SNP markers
Source: Front Genet. 2022 Aug 24;13:907267. doi: 10.3389/fgene.2022.907267 (PMC9467640; doi:10.3389/fgene.2022.907267)
Supplement: Supplementary file 1 [file Presentation1.PPTX]

## Slide 1
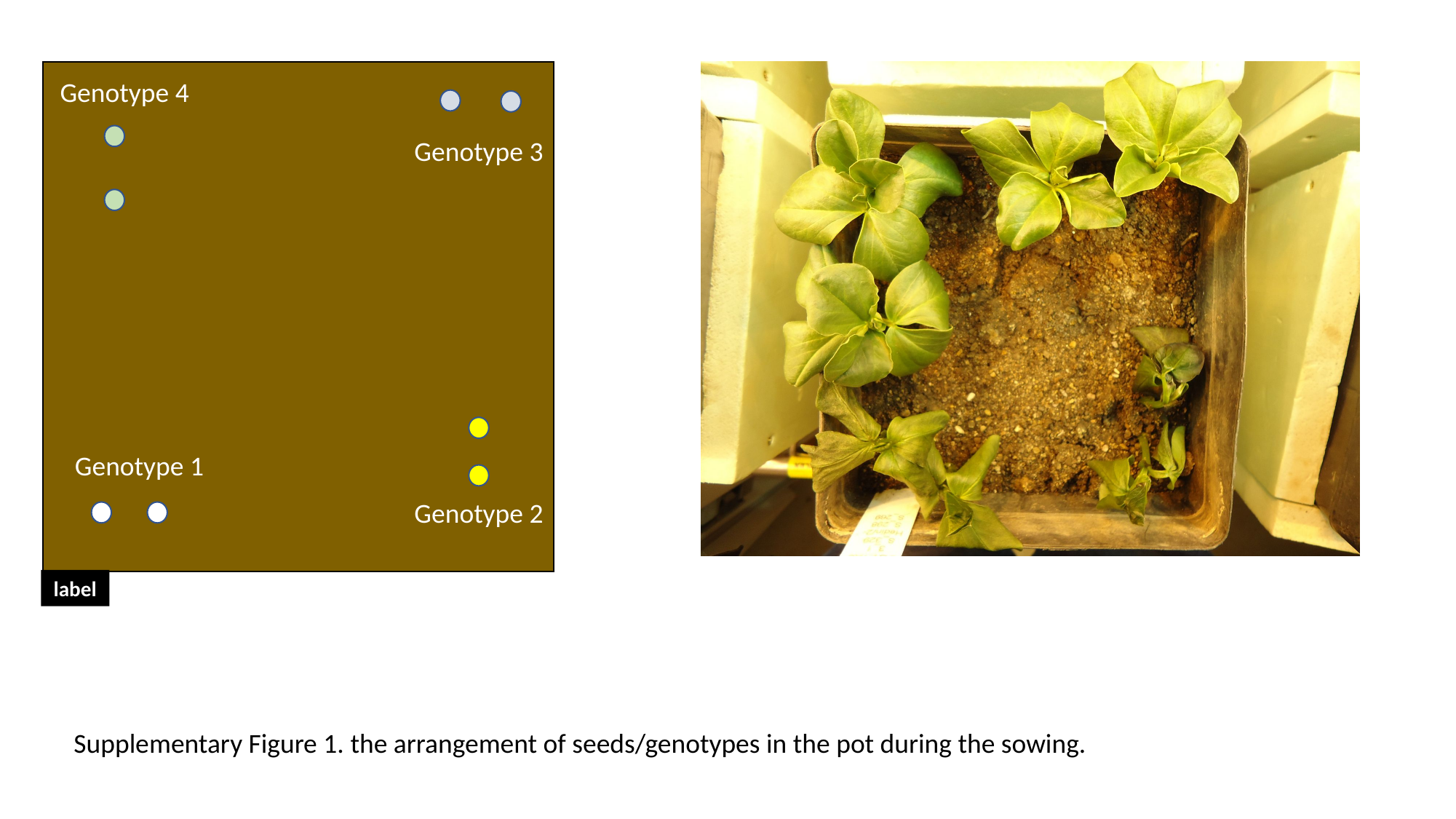

Genotype 4
Genotype 3
Genotype 1
Genotype 2
label
Supplementary Figure 1. the arrangement of seeds/genotypes in the pot during the sowing.

## Slide 2
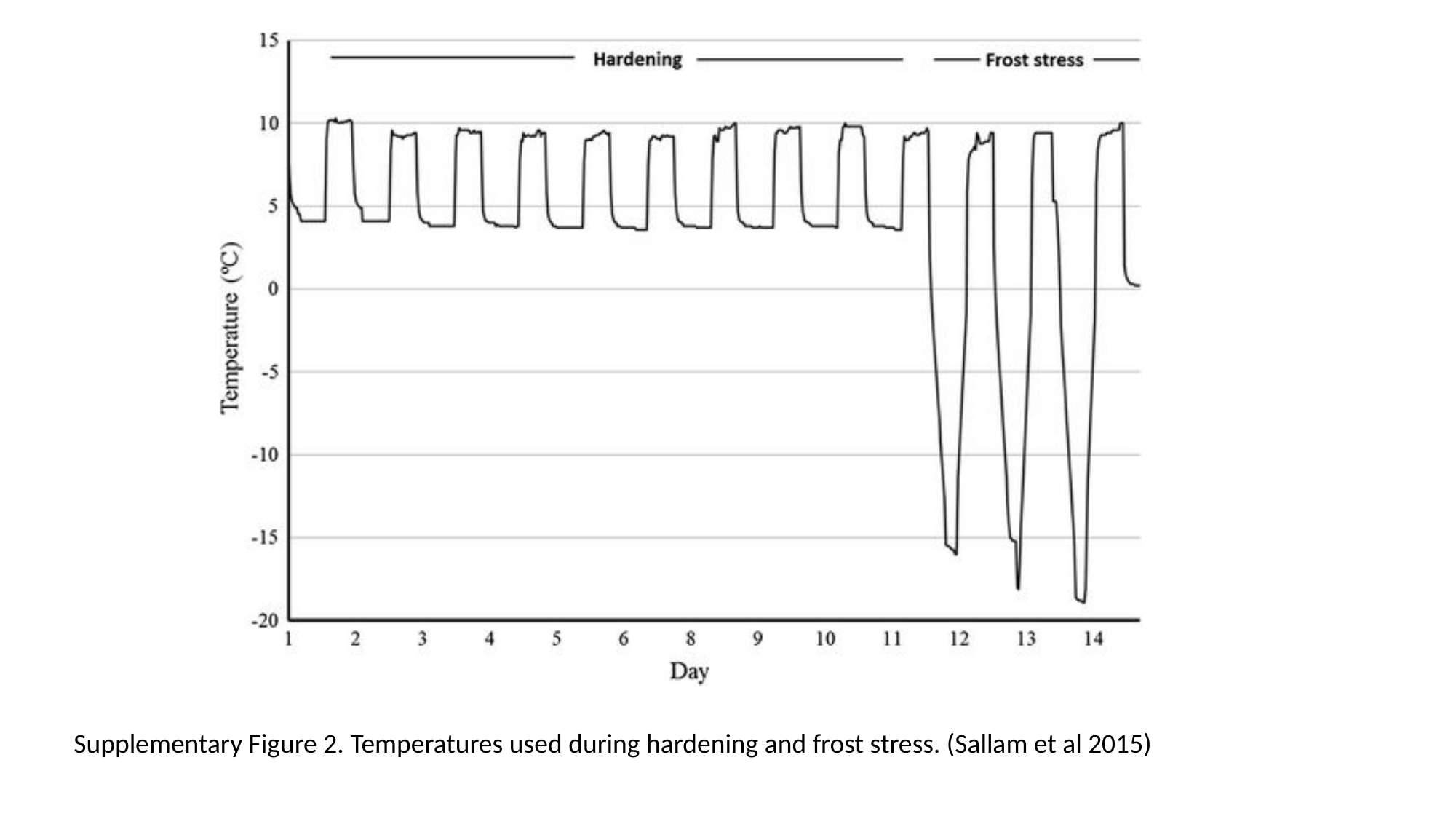

Supplementary Figure 2. Temperatures used during hardening and frost stress. (Sallam et al 2015)

## Slide 3
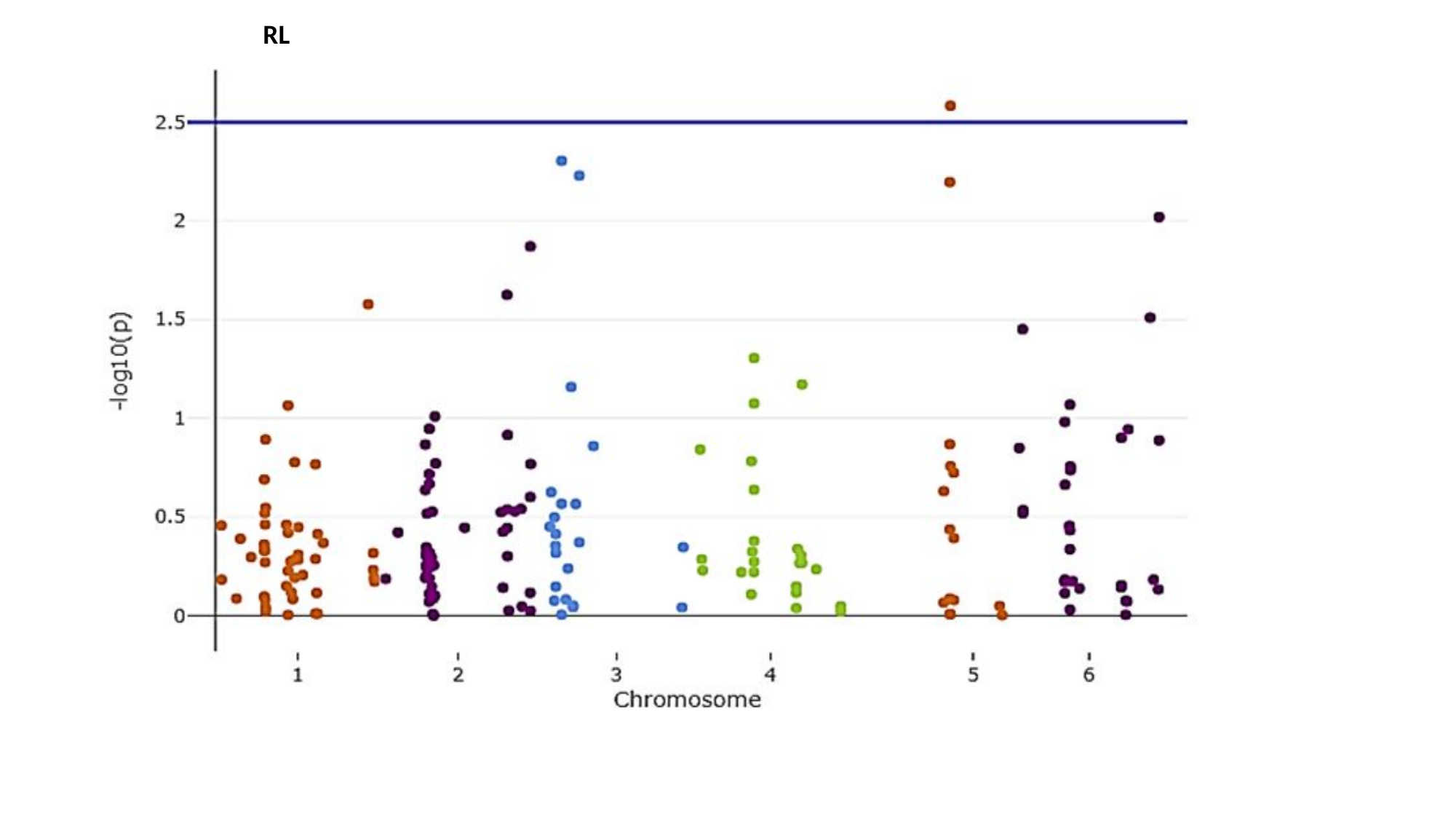

RL

## Slide 4
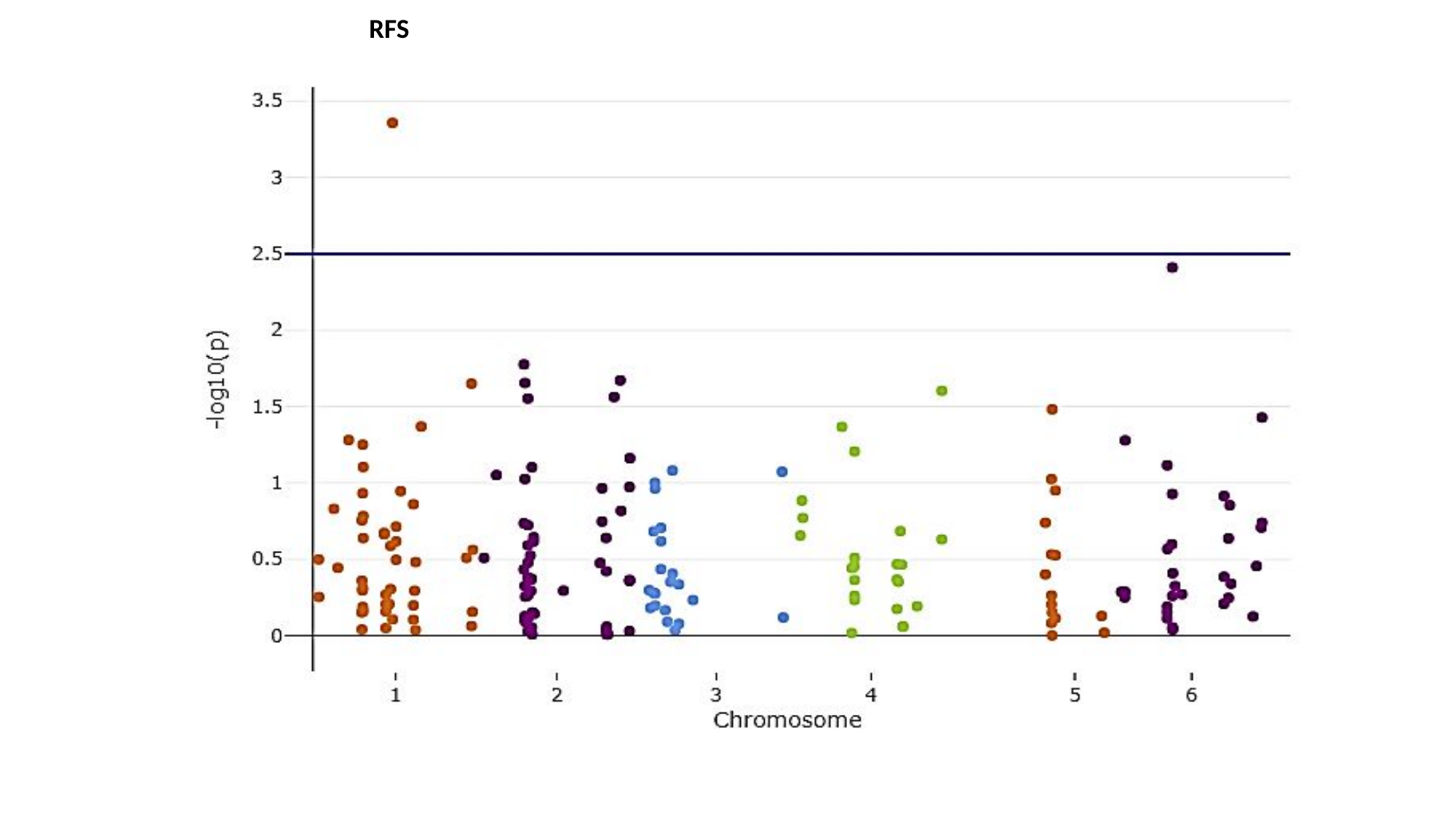

RFS

## Slide 5
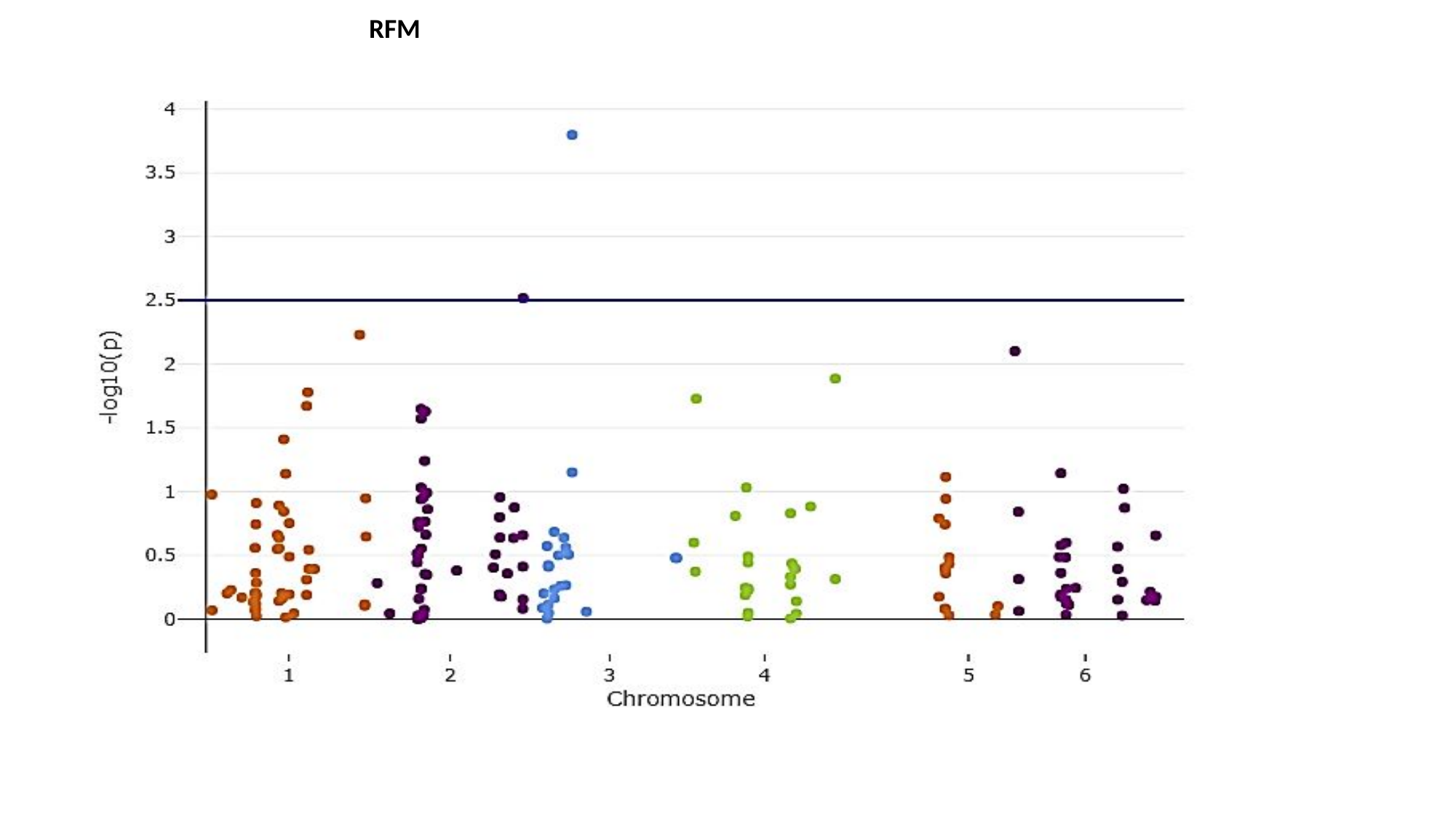

RFM

## Slide 6
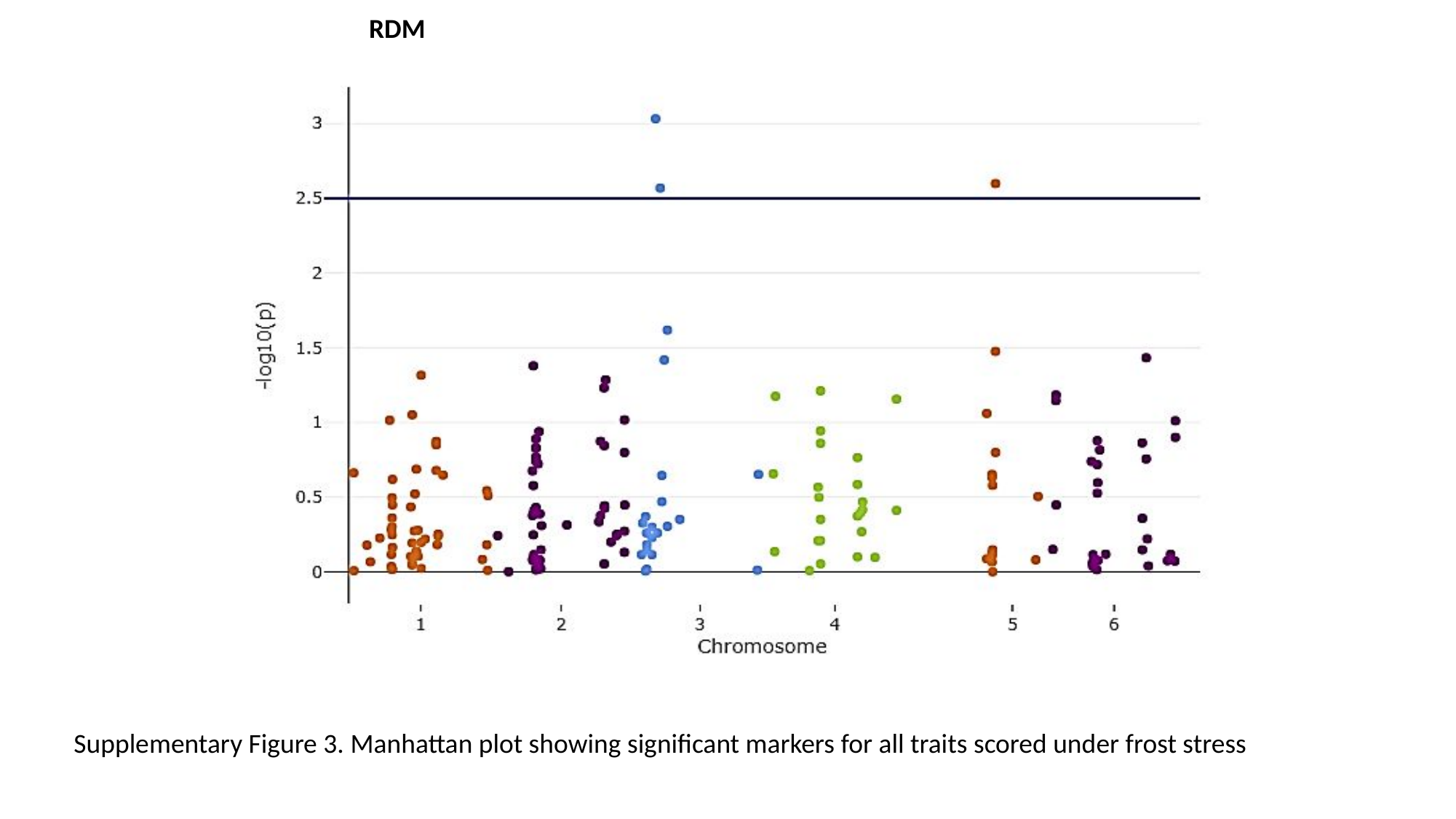

RDM
Supplementary Figure 3. Manhattan plot showing significant markers for all traits scored under frost stress
